# Muddying the Waters: A New Area of Concern for Drinking Water Contamination in Cameroon

**DOI:** 10.3390/ijerph111212454

**Published:** 2014-11-28

**Authors:** Jessica M. Healy Profitós, Arabi Mouhaman, Seungjun Lee, Rebecca Garabed, Mark Moritz, Barbara Piperata, Joe Tien, Michael Bisesi, Jiyoung Lee

**Affiliations:** 1 Division of Environmental Health Sciences, the Ohio State University, Columbus, OH 43210, USA; E-Mails: jessicamorganhealy@gmail.com (J.M.H.P.); bisesi.12@osu.edu (M.B.); 2 Department of Environmental Sciences, University of Maroua, Maroua BP 46, Far North Region, Cameroon; E-Mail: mouharabi@yahoo.fr; 3 Department of Food Science and Technology, the Ohio State University, Columbus, OH 43210, USA; E-Mail: lee.5178@osu.edu; 4 Department of Veterinary Preventive Medicine, the Ohio State University, Columbus, OH 43210, USA; E-Mail: garabed.1@osu.edu; 5 Department of Anthropology, the Ohio State University, Columbus, OH 43210, USA; E-Mails: moritz.42@osu.edu (M.M.); piperata.1@osu.edu (B.P.); 6 Department of Mathematics, the Ohio State University, Columbus, OH 43210, USA; E-Mail: tien.20@osu.edu

**Keywords:** diarrhoeal illness, drinking water quality, microbial source tracking, drinking water storage, drinking water distribution

## Abstract

In urban Maroua, Cameroon, improved drinking water sources are available to a large majority of the population, yet this water is frequently distributed through informal distribution systems and stored in home containers (*canaries*), leaving it vulnerable to contamination. We assessed where contamination occurs within the distribution system, determined potential sources of environmental contamination, and investigated potential pathogens. Gastrointestinal health status (785 individuals) was collected via health surveys. Drinking water samples were collected from drinking water sources and *canaries*. *Escherichia coli* and total coliform levels were evaluated and molecular detection was performed to measure human-associated faecal marker, HF183; tetracycline-resistance gene, *tet*Q; *Campylobacter* spp.; and *Staphylococcus aureus*. Statistical analyses were performed to evaluate the relationship between microbial contamination and gastrointestinal illness. *Canari* samples had higher levels of contamination than source samples. HF183 and *tet*Q were detected in home and source samples. An inverse relationship was found between *tet*Q and *E. coli*. Presence of *tet*Q with lower *E. coli* levels increased the odds of reported diarrhoeal illness than *E. coli* levels alone. Further work is warranted to better assess the relationship between antimicrobial-resistant bacteria and other pathogens in micro-ecosystems within *canaries* and this relationship’s impact on drinking water quality.

## 1. Introduction

In 2011, diarrhoeal illness was responsible for the death of 1.9 million people [1]. A majority of these deaths occurred in children under the age of five living in developing countries [2]. Due in part to their underdeveloped immune systems, diarrhoea kills more children annually than malaria and is the second leading cause of mortality behind pneumonia [2,3].

In addition to improving sanitation, there are two main ways to reduce the amount of microbial contamination in drinking water and, thus, reduce the incidence of diarrhoeal disease in developing countries: (1) increasing availability and access to clean water suitable for drinking; and, (2) providing improved methods of drinking water storage that prevent recontamination in the home [4,5,6]. Improving the quality and availability of drinking water is helpful in reducing diarrhoeal disease incidence, but if water is then contaminated during transportation and storage, diarrhoeal disease transmission is still possible. While there has been some debate on which side of the improved source water-improved home drinking water equation results in a greater decrease in diarrhoeal disease [7,8,9,10,11,12,13], all sides agree that tackling the problem from both ends is the most desirable approach. This is especially true for preventing diarrhoea in children under the age of five who have not yet developed immunity to the naturally circulating human pathogens that are present in the drinking water storage containers in their homes [13].

**Figure 1:**
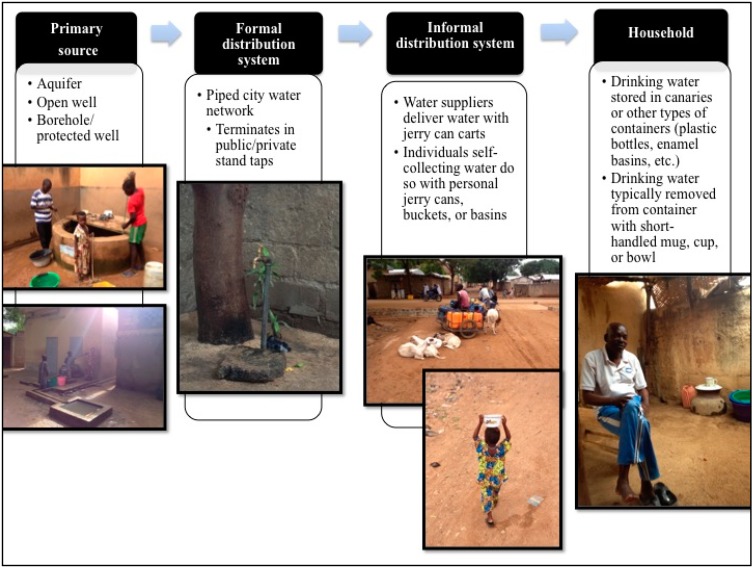
Flowchart of the drinking water delivery chain in Maroua including both formal and informal distribution systems.

This study was conducted in Maroua, a regional capital of Cameroon. Maroua was chosen because recent epidemiological information (*i.e.*, cholera incidence rates) collected during the 2009–2011 cholera outbreak allowed for identification of neighbourhoods that had high, middle, and low incidence rates of diarrhoeal illness. Home to over 300,000 people, Maroua also provided an opportunity to explore drinking water quality in an urban environment where a large percentage of people obtain their drinking water through an informal distribution system of water suppliers who transport the water from city taps to homes using jerry cans (Figure 1), a situation that is common throughout the developing world [14].

Previous drinking water quality studies have been conducted in the Far North Region and in other regions of Cameroon. Previous studies have mostly focused on well water quality [15,16], quality of ground and surface waters [17], general associations between access to drinking water and diarrhoeal illness [18], or the management of household drinking water treatment [19]. Despite the fact that Maroua is a rapidly growing urban centre [20] of great importance to the greater Chad Basin region, the majority of drinking water and gastrointestinal health studies carried out to date have been in the more southern regions of the country [17,18,19,21,22,23]. To the best of our knowledge, this was the first study to investigate the associations between diarrhoeal disease and the drinking water quality within the formal and informal drinking water delivery system in addition to canaries within the city of Maroua. The aims of this study were: (1) to investigate microbial contamination of drinking water along the water delivery system within the city; (2) to identify the potential sources of the microbial contamination from the general environment using microbial source tracking; (3) to examine the relationship between water quality and reported gastrointestinal illnesses; and, (4) test for the presence of antibiotic resistant microbes in drinking water.

## 2. Experimental Section

### 2.1. Study Site

In order to achieve these aims, health surveys were administered to residents of the study households, and drinking water samples were collected from home storage containers (HSC) and drinking water sources of households located in four different Maroua neighbourhoods between 1 June and 1 August 2013. Sampling coincided with the beginning of the rainy season, the traditional peak season of cholera outbreaks. Neighbourhoods were selected based on their relative cholera incidence rates (one high, two medium, one low) during the 2009–2011 epidemic. The number of households surveyed in each neighbourhood was based on the relative proportion that the neighbourhood contributed to Maroua’s overall population. A map of each neighbourhood was printed and sectioned into numbered quadrants using a 2 cm × 2 cm grid. The quadrants were then randomly selected by drawing numbered pieces of paper and one household within each quadrant was surveyed.

### 2.2. Health Survey

The health survey was administered in a total of 120 households. The survey included questions on household demographics, as well as reported health and drinking water handling behaviours of the individual members of the household. For children too young to respond, caretakers (usually the mother) answered for them. In terms of health, individuals were asked if they had experienced any of the following gastrointestinal symptoms during the 30 days prior to the survey: diarrhoea, bloody diarrhoea, stomach cramps, vomiting, nausea, and/or fever. Participants were asked how many days the symptom(s) had lasted, if they had received treatment at a hospital and/or had been under a doctor’s care for any reason, and for how many days they received care or treatment. Each survey lasted 20–50 min, depending on family size.

Due to low literacy rates in the study area, informed consent was verbally obtained before proceeding with each survey and documented through the full completion of the survey. The guardian(s) of children enrolled in the study also provided verbal, informed consent for the release of their children’s information and at least one guardian was present during each survey. Each home and individual household member was assigned unique identification codes to ensure anonymity. Participants were made aware that they were free to stop the survey at anytime, for any reason and without penalty. Research involving survey of human subjects (including verbal consent) was approved from The Ohio State University Institutional Review Board (IRB 2010B004 and Amendment 032013) and permission to perform fieldwork was granted from the local Cameroon Ministry of Health, local délégués, and traditional community leaders.

### 2.3. Drinking Water Sample Collection

**Figure 2:**
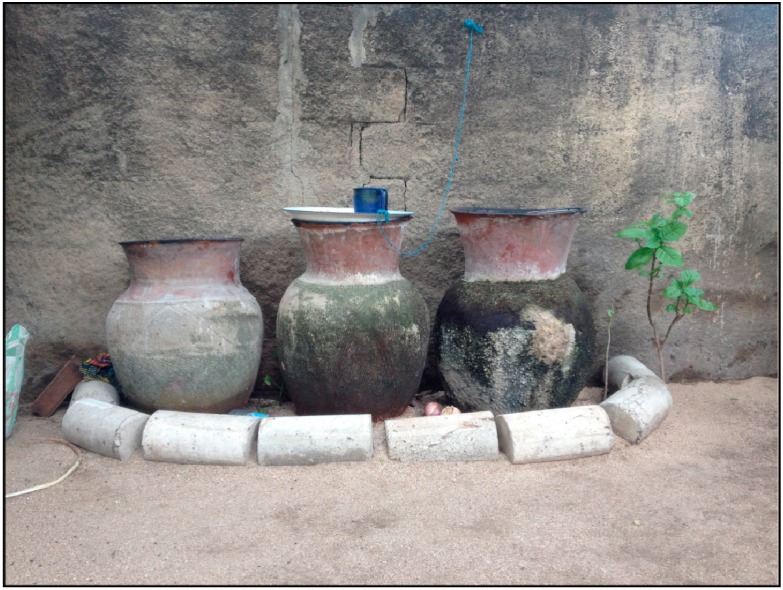
*Canaries* in a household’s compound being used for drinking water storage.

Drinking water samples were collected from households that had water available (*n* = 60). Between 300 and 750 mL of water were taken directly from the family’s drinking water storage container, which was usually a traditional, wide-mouth clay jar (*canari* in French) used to allow the water to “breathe” and stay cool in a hot Sahelian climate (Figure 2). Home water storage is necessary due to frequent, unpredictable rationing of city tap water. When accessible, samples were also taken from the originating source where drinking water was procured (*n* = 28), which was usually a public tap, but also included water distributers’ jerry cans, open wells, and a community borehole. Out of the total 86 drinking water samples collected, 25 home samples were paired to a matching source. All samples were collected using single, sterile 800 mL, pre-labeled Whirl-Pak bags (Nasco, Fort Atkinson, WI, USA). The sampling bags were filled with water using the same technique that household members typically used when handling their drinking water. The drinking water samples were then immediately placed in a cooler and transported to a field laboratory where chemical and biological analyses were conducted.

#### 2.3.1. Water Quality Measurements

Chemical quality of the water (total chorine, mg/L; free chlorine/bromine, mg/L; pH; total alkalinity, mg/L; total hardness, mg/L of CaCO_3_; and cyanuric acid, mg/L) was evaluated using a six-way paper-testing strip (Kokido Development Ltd., Hong Kong). Biological quality was initially evaluated in the field using Coliplates^®^ (Bluewater Biosciences, Inc. Ontario, ON, Canada), which measured the most probable number of colony forming units (CFU)/100 mL of both total coliforms and *E. coli* [24].

#### 2.3.2. Molecular Detection

Aliquots of the same water sample were then filtered through a sterile 0.45 micron Millipore size membrane filter (IsoporeTM Membrane Filters, Millipore, Tullagreen, Cork, Ireland), placed in a sterile 2 mL test tube, and immediately frozen at −20 °C. The filters were transported back to the laboratory located at The Ohio State University, College of Public Health, Division of Environmental Health Sciences in the United States on ice for further analyses.

To evaluate the potential sources of microbial contamination, the human-specific *Bacteroides* genetic marker, HF183, was targeted to identify human faecal contamination [25]; *Campylobacter* spp. was determined to assess potential livestock/wildlife faecal contamination by targeting the 16S rRNA gene of *C. jejuni*, *C. coli* and *C. lari* [26]; and *Staphylococcus aureus* was detected to assess potential contact of drinking water with human skin (*i.e.*, hand contact) by targeting *nuc* gene [27]. As this was a pilot project for a larger study, resource constraints only allowed for the assessment of antibiotic resistance to one class of antibiotics. Tetracycline was selected as it is one of the most commonly used antibiotics in both human and veterinary medicine both in the area [28,29,30] and globally [31]. Although there are numerous genes that code for tetracycline resistance [32], due our limited resources, only *tet*Q was targeted to assess the presence of antibacterial-resistant bacteria and also, potentially, human-and livestock-associated faecal contamination. This specific gene was targeted as it has been documented in previous studies to be the gene with the highest detection frequency in wastewater influent [31], was found to be one of the tetracycline resistance genes in greatest abundance in surface waters associated with livestock production [33], and has been documented as having the ability to transfer horizontally between bacteria native to human and ruminant gastrointestinal tracts [28]. Further information regarding PCR analysis methods can be reviewed in the supplementary material.

### 2.4. Statistical Analyses

Statistical analyses were performed using Microsoft Excel 2008 (Release ver. 12.3.6 Microsoft^®^ Excel^®^) and Stata 13.1 (StataCorp Lp, College Station, TX, USA) software. Due to the non-normal distribution of the data, Mann-Whitney U tests were used to compare contamination levels between source and home samples. Spearman Rank-Order analysis and adjusted odds ratios were used to evaluate the relationships among water quality parameters and to evaluate the relationship between gastrointestinal illness and the quality of stored drinking water, respectively. During the calculation of the odds ratios, the null hypothesis assumed was that the microbial quality of stored drinking water would have no effect on the gastrointestinal health of individuals in the household. The alternative hypothesis was that there would be some effect, either protective (OR less than 1) or that the odds of reporting a gastrointestinal event would be higher (OR greater than 1) based on the presence of microbial contamination.

## 3. Results and Discussion

### 3.1. Health Survey Results

The descriptive demographic statistics are summarized in Table 1. Interviews were conducted in 120 households (*n* = 785 individuals). Average household size was 6.7 members, with several homes having more than 20 members. As in many developing countries, the age-structure of the population was young; 14% of individuals were under five years and 52% were under age 18 years. The median age was 17 years and average age was 21 years.

The top three gastrointestinal illness (GI) symptoms reported during the survey were stomach cramps, diarrhoea and fever (Table 2). On average, stomach cramps were reported to last 6 days (range 1–60 days); diarrhoea, 6 days (1–28); and fever, 8 days (1–60). 11% of the total survey population reported receiving care at a hospital (average duration 2.1 days) and 16% reported being under a doctor’s care (average 6.6 days).

**Table 1:** Descriptive statistics of demographic information obtained from heath surveys.

Demographic Category	Category of Measure	Number	% of Total Study Population (Individuals)
Number of Households Surveyed	Total	120	-
Number of Individuals Included on Surveys	Total	785	-
Age	Mean	21.0 years	-
	Median	17 years	-
	Range	3 days-100 years	-
Number less than 5 years	Total	97	13.8%
Number between 5-18 years	Total	286	40.6%
Number less than 18 years	Total	367	52.1%
Number between 19-60 years	Total	301	42.8%
Number over 60 years	Total	101	14.3%
Gender	Total Males	385	49.0%
	Total Females	400	51.0%
Household Size	Mean	6.7 people	-
	Median	6.5 people	-
	Range	2–25 people	-
Religion	Total Muslim	640	81.5%
	Total Christian	143	18.2%
	Total Animist/Other	2	0.3%
Number of different ethnic groups reported	Total	44	-

**Table 2:** Number and percentage of individuals who reported gastrointestinal illness symptom(s) during the health survey; overall, and by age class and gender.

	Overall	(%)	<2 Years	(%)	<5 Years	(%)	5–18	(%)	19–60	(%)	61+	(%)
Diarrhoea ^1^	98	12.5	16	44.4	23	23.71	34	11.88	31	10.29	1	5.2
*Males*	41	41.8	7	43.7	11	47.8	12	35.3	11	35.5	1	100
*Females*	57	58.2	9	56.3	12	52.2	22	64.7	20	64.5	0	0
Bloody diarrhoea	9	1.14	1	2.78	2	2.06	5	1.74	1	0.33	0	0
*Males*	4	44.4	1	100	2	100	2	40.0	0	0	-	-
*Females*	5	55.6	0	0	0	0	3	60.0	1	100	-	-
Stomach ^2^ cramps	131	16.7	4	11.11	11	11.34	55	19.23	50	16.61	3	1.57
*Males*	44	33.6	1	25	5	45.5	16	29.1	14	28.0	1	33.3
*Females*	87	66.4	3	75	6	54.5	39	70.9	36	72.0	2	66.7
Vomiting	25	3.1	2	5.56	2	2.06	8	2.79	9	2.99	0	0
*Males*	14	56.0	1	50	1	50	5	62.5	4	44.4	-	-
*Females*	11	44.0	1	50	1	50	3	37.5	5	55.6	-	-
Nausea	34	4.3	0	0	0	0	13	4.54	17	5.64	0	0
*Males*	12	35.3	-	-	-	-	4	30.8	6	35.3	-	-
*Females*	22	64.7	-	-	-	-	9	69.2	11	64.7	-	-
Fever ^3^	109	13.9	3	8.33	6	6.1	43	15.03	43	14.28	3	15.78
*Males*	58	53.2	3	100	6	100	22	51.2	19	44.2	2	66.7
*Females*	51	46.8	0	0	0	0	21	48.8	24	55.8	1	33.3

^1^ The third most reported symptom. ^2^ The number one most reported symptom. ^3^ The second most reported symptom. Note: 80 people were unsure of their age and were removed for purposes of the age-related breakdown. They remain included in the overall statistics.

Reported gastrointestinal symptoms were higher for the younger age ranges: 55% of individuals 5–18 years (158/286) and 45% of individuals under the age of 5 years (44/97) reported to have had at least one GI symptom in the prior month. Over 16% percent (16/98) of individuals who had experienced diarrhoea were under the age of 2, despite this age group only making up only 5% of the overall population (36/705).

### 3.2. Sources and Storage of Drinking Water

Overall, 61% of surveyed households reported receiving their drinking water through a water supplier who transports water from a public tap or private water seller using plastic jerry cans loaded on a pull cart and delivers the water to their home; 20% had a private tap in their homes or compound; 13% bought their water from a neighbour using their own jerry cans; 3% regularly got their drinking water from a well; and 2% got their drinking water from a community borehole. Although a majority of households received their drinking water from a supplier, the supplier was not always available in order to obtain a sample from his jerry can. In these cases, the family directed us to the public tap source where their water supplier regularly obtained their water and a sample was taken from that tap. This resulted in the collection of 18 source samples directly from public/private taps, three from water suppliers’ jerry cans, three from open wells, and one from a borehole.

89% of households surveyed reported storing their drinking water at home in *canaries*. This included households with private taps within their compounds. Households who did not store their water in *canaries* generally used plastic bottles, buckets, and/or basins. 93% of households reported that they always or usually kept their drinking water storage containers (all types) covered. 73% said that they refilled the container every day, 22% at least every other day, and 1% every 3 or more days.

### 3.3. Drinking Water Quality

#### 3.3.1. Chemical

Chemical analyses of all drinking water samples (both home and source water) revealed little detectable chlorine (average 0.23 mg/L of total chlorine and 0 mg/L of free chlorine). The average pH of all samples was 7.0. Average total alkalinity was 157 mg/L and the total hardness CaCO_3_ was 75.9 mg/L. Other than free chlorine levels, there were no statistically significant differences in chemical quality between home and source samples (Table 3).

**Table 3:** Comparison of water quality from the water samples collected at drinking water sources and homes (source, *n* = 25; home, *n* = 59).

Parameter	Statistic	Source	Home	*p*-Value (Mann-Whitney U Test)
Total Coliforms (CFU/100 ml)	Mean ^a^	3.2 × 10^2^	1.7 × 10^3^	0.0001 ^b^
	Median	2.2 × 10^1^	2.4 × 10^3^	-
	Range	2.2 × 10^1^–2.4 × 10^3^	2.2 × 10^1^–2.4 × 10^3^	-
*E. coli* (CFU/100 ml)	Mean	6.8 × 10^1^	5.5 × 10^2^	0.0004 ^b^
	Median	4.0 × 10^0^	1.6 × 10^2^	-
	Range	2.2 × 10^1^–8.6 × 10^2^	2.2 × 10^1^–2.4 × 10^3^	-
Total Chlorine (mg/L)	Mean	0.4	0.1	0.1067
	Median	0	0	-
	Range	0–3	0–5	-
Free Chlorine/Bromine (mg/L)	Mean	0.11	0.01	0.0390 ^b^
	Median	0	0	-
	Range	0–0.75	0–0.5	-
pH	Mean	6.9	7.0	0.3118
	Median	6.8	7.2	-
	Range	6.8–7.6	6.8–8.0	-
Total Alkalinity (mg/L)	Mean	148.6	161	0.4808
	Median	120	180	-
	Range	80–240	80–240	-
Total Hardness (mg/L CaCO_3_)	Mean	64.8	80.8	0.5787
	Median	0	100	-
	Range	0–500	0–1000	-

^a^ All means reported are arithmetic. ^b^ Home and source values are statistically significant, corresponding *p*-values listed.

#### 3.3.2. Microbial

Microbial analysis of all drinking water samples (both home and source water) revealed a high level of bacterial contamination that surpassed the World Health Organization’s “no action required” threshold of <1 CFU/100 mL for *E. coli* in drinking water [34]. *In-situ* analysis showed that the average total coliform count for all samples (*i.e.*, both home and source samples) was 1.27 × 10^3^ CFU/100 mL (median value = 1.4 × 10^3^) and the average *E. coli* count was 4.3 × 10^2^ CFU/100 mL (median value = 7.8 × 10^1^). When home and source samples were separated, home samples had statistically significant higher levels of coliforms (*p* = 0.0001) and *E. coli* (*p* = 0.0004) (CFU/100 mL coliform average 1.7 × 10^3^, median 2.4 × 10^3^; CFU/100 mL *E. coli* average 5.5 × 10^2^, median 1.6 × 10^2^) compared to source samples (CFU/100 mL coliform average 3.2 × 10^2^, median 2.2 × 10^1^; CFU/100 mL *E. coli* average 6.8 × 10^1^, median 4 × 10^0^) (Table 3).

**Figure 3:**
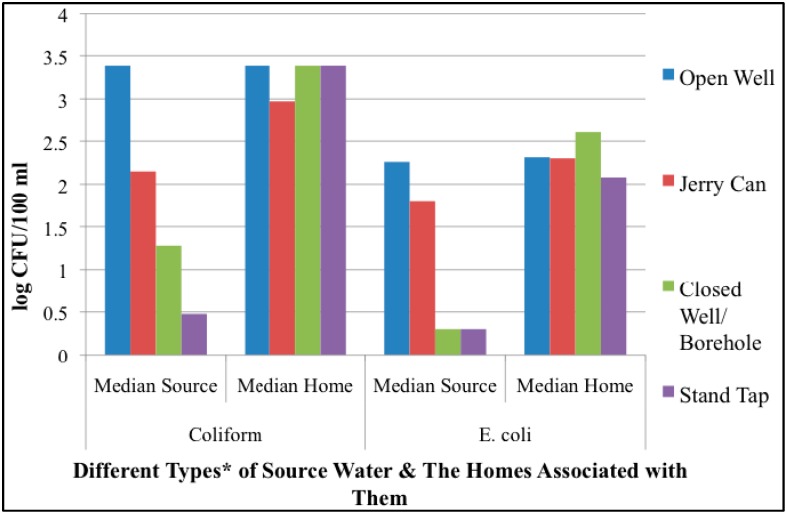
Total coliforms and *E. coli* median concentration values from different types of drinking water sources and home storage container.

Most source samples (18/25) were taken from improved drinking water sources (*i.e.*, stand taps) but most of the bacterial contamination in source samples were detected from the few non-improved sources that were collected (e.g., open wells; 3/25). Because fewer non-stand tap sources (*n* = 7) were able to be collected and because their microbial quality greatly skewed the data, they were removed from subsequent analyses. Upon removing these non-tap sources from the analysis, the difference between paired home and tap source water quality samples was even greater in terms of *E. coli* and coliform levels (Home: mean *E. coli* level = 5.51 × 10^2^ CFU/100 mL, median *E. coli* level = 1.6 × 10^2^; mean total coliform = 1.03 × 10^3^, median coliform = 2.4 × 10^3^. Taps: mean *E. coli* level = 5.8 × 10^1^, median *E.coli* level = 2 × 10^0^; mean total coliform level = 1.77 × 10^2^, median total coliform level = 4 × 10^0^. *E. coli*, *p =* 0.0001; total coliform, *p* = 0.001). Yet it is important to note that although water collected directly from taps exhibited lower concentrations of both total coliform bacteria and *E. coli*, non-tap sources collected indicated that once the drinking water began moving through the informal distribution system (e.g., into water suppliers’ jerry cans), microbial contamination levels rose substantially for both total coliforms and *E. coli*. This increase in microbial contamination suggested that receiving water from an improved source had only a small impact on the quality of the water at the point of use, the home (Figure 3). Note, due to the non-normal distribution of contamination levels and small sample sizes of non-tap sources, the median concentration values have been displayed.

* There was only 1 sample taken from a closed well/borehole. 3 from open wells, 3 from jerry canes and 18 from stand taps.

#### 3.3.3. Molecular Detections

qPCR analysis detected tetracycline-resistant gene copies (*tet*Q) and human faecal marker gene copies, HF183; in 19% (*n* = 13) and 7% (*n* = 5) of total samples, respectively (Table 4). *Campylobacter* spp., a common waterborne pathogen of zoonotic origin, and *S. aureus*, a common part of the natural flora of human skin, were also detected in 12% (*n* = 8) and 19% (*n* = 13) of all water samples, respectively. With the exception of *tet*Q and HF183, home samples had substantially higher positive test frequencies of all other bacteria assayed compared to source samples. This is especially true of *S. aureus* and *Campylobacter* positive samples, which were found in twice to three times higher percentages in homes *versus* source samples.

### 3.4. Reported Gastrointestinal Illness & Drinking Water Quality

Based on the results of the health survey, individuals were divided into four categories using GI symptoms in the prior 30 days: (1) non-case; (2) diarrhoea case (*i.e.*, any individual who reported experiencing diarrhoea); (3) highly confirmed case of gastrointestinal illness with bloody diarrhoea (HCGI-BD; any individual who reported experiencing bloody diarrhoea, stomach cramps, and fever); or (4) highly confirmed case of gastrointestinal illness without bloody diarrhoea (HCGI-NBD; any individual who reported experiencing non-bloody diarrhoea, stomach cramps, and fever).

**Table 4:** Microbial contamination found in water samples from stand tap water sources and all home storage containers.

Genetic Marker or Bacteria	Positive Source º	Positive Home ª	Total Positives
	*n (% Positive source samples)*	*n (% Positive home samples)*	*n (% Of total samples)*
*E. coli* ^*,Υ^	9 *(50)*	52 *(87)*	61 *(87)*
*tet*Q ^ŧ^	5 *(28)*	8 (*13)*	13 *(19)*
*S. aureus* ^ŧ^	1 *(6)*	12 *(20)*	13 *(19)*
*Campylocbacter* ^ŧ^	1 *(6)*	7 *(12)*	8 *(12)*
HF183 ^ŧ^	1 *(6)*	4 *(7)*	5 *(7)*

º Source samples only include source samples taken from stand taps (*n* = 18). ª Includes all home samples regardless of source type (*n* = 60). ^*^ Mesaured in CFU/100 mL and includes samples above the ColiPlate limit of detection. ^ŧ^ Measured in gene copies/100 mL and includes samples above qPCR limit of detection. ^Υ^ 2 samples were lost during DNA extraction, thus the denominator for *E.coli* samples is 88 instead of 86.

Spearman Rank-Order analyses were used to measure the relationship between the following home drinking water quality parameters: number of HF183 gene copies/100 mL; number of *tet* Qgene copies/100 mL; and concentration of *E. coli* CFU/100 mL. *tet*Q had a statistically significant inverse relationship both with *E. coli* levels (Rho = −0.31, *p* = 0.001) and with HF183 (Rho = −0.11, *p* = 0.003). Initial statistical analyses did not demonstrate a statistically significant relationship between *Campylobacter*, *S. aureus* and gastrointestinal illness, thus these parameters were not included in further analyses. Mann-Whitney U tests comparing *tet*Q and *E. coli* levels present in the HSC of cases and non-cases demonstrated a significant difference only in the average level of *tet*Q for all three GI-illness categories (Table 5).

Two rounds of odds ratios were then performed in order to further measure the association between drinking water quality at the point of use (*i.e.*, within *canaries*) and reported gastrointestinal illness. The first round was performed on each of the following three classes (diarrhoea, HCGI-BD, and HCGI-NBD) and with the following drinking water quality parameters: *E. coli* quartiles, presence of HF183, and presence of *tet*Q. Simple presence/absence of *tet*Q and HF183 genes copies were used in order to include all individuals who had any detectable amount of HF183 or *tet*Q in their drinking water (Table 6). Due to their non-normal distribution and in order to better view the risk associated with varying levels of potential faecal contamination, the *E. coli* concentration measures were divided and reported as quartile values. Because diarrhoeal disease symptoms were more heavily reported among younger individuals, the odds ratios were adjusted for age.

**Table 5:** Results of Mann-Whitney U tests of *E. coli* and *tet*Q concentrations between different types of gastrointestinal illness (diarrhoea, HCGI-BD, HCGI-NBD).

Gastrointestinal Illness Class (*n* = Cases/*n* = Non-Cases) ^1^	Water Quality Parameter Medians & ( *Means*)	Cases	Non-Cases ^Y^	*p*-Value
Diarrhoea ^2^	*tet*Q (gene copies/100 mL)	7.5 (*547.9*)	7.5 (*278.5*)	0.0032 ^*^
(52/347)	*E. coli* (log CFU/100 mL)	1.9 *(2.0)*	2.2 *(2.1)*	0.9405
HCGI-BD ^3^	*tet*Q (gene copies/100 mL	2310.6 *(2310.6)*	7.5 *(307.2)*	0.0194 ^*^
(1/398)	*E. coli* (log CFU/100 mL)	1.11 *(1.1)*	2.3 *(2.1)*	0.2792
HCGI-NBD ^4^	*tet*Q (gene copies/100 mL	7.5 *(773.2)*	7.5 *(290.5)*	0.0021 ^*^
(18/381)	*E. coli* (log CFU/100 mL)	1.6 *(1.8)*	2.3 *(2.1)*	0.2377

^1^ Total sample for surveyed individuals was 785, but water quality was only available for 399 individuals; ^2^ Case = any individual who reported experiencing diarrhoea during prior 30 days; ^3^ Case = any individual who reported experiencing bloody diarrhoea, stomach cramps & fever during prior 30 days; ^4^ Case = any individual who reported experiencing non-bloody diarrhoea, stomach cramps & fever during prior 30 days; ^Y^ Non-cases = any individual who did not meet the definitions defined above; ^*^ Statistically significant.

**Table 6:** Adjusted odds ratios for diarrhoea, HCGI-BD & HCGI-NBD associated with various exposures levels to *E. coli*, HF183, or *tet*Q.

Genetic Marker or *E. coli* Quartile (Range log CFU/100 mL)	Diarrhoea		HCGI with Bloody Diarrhoea		HCGI with Non-Bloody Diarrhoea	
	aOR ^1^ (95% CI)	(*p*)	aOR (95% CI)	(*p*)	aOR (95% CI)	(*p*)
*E. coli* Quartile ^†^ (0.30–1.23) ^‡^	1.15 (0.57–2.31)	0.13	N/A ^2^	-	1.62 (0.61–4.92)	0.35
*E. coli* Quartile (1.24–2.26)	1.92 (0.93–3.42)	0.05	N/A	-	1.72 (0.63–5.08)	0.30
*E. coli* Quartile (2.27–2.93)	0.08 (0.93–3.42)	0.05	N/A	-	N/C	-
*E. coli* Quartile (2.92–3.38)	1.43 (0.78–2.98)	0.31	N/A	-	1.01 (0.32–3.22)	0.95
HF183	0.92 (0.16–3.18)	0.91	N/C ^3^	-	2.57 (0.5–12.07)	0.21
*tet*Q	2.48 (1.3–5.16)	0.01 ^*^	N/A	-	3.01 (1.02–8.89)	0.04 ^*^

^1^ Odds ratio adjusted for age using Mantel-Haenszel method and null and alternative hypotheses are as follows, H_0_: OR = 1 *versus* H_1_: OR ≠ 1; ^2^ Not Available. There were only 3 cases of HCGI with bloody diarrhoea and in certain cases, ORs could not be calculated due to zero cell counts; ^3^ No cases exposed in this quartile; † *E. coli* concentration divided into quartiles to better demonstrate the varying risk with different contamination levels; ^‡^ inclusive cut-offs for each quartile; ^*^ Statistically significant confidence intervals above an OR of 1 or “no effect”.

Guided by the results of the Spearman Rank-Order analysis (inverse relationship between *tet*Q and *E. coli*) and the results of the first round of odds ratio calculations (increased odds of reporting gastrointestinal illness with the presence of *tet*Q in home drinking water), a second round of odds ratios was conducted using the same three case classes and placed them in four different water quality groups created based on the presence of *tet*Q and varying *E. coli* levels in HSC drinking water (Table 7). The highest statistically significant odds ratios occurred between diarrhoea and NCGI-NBD cases that had *E. coli* levels below the 1st quartile and *tet*Q present in their drinking water. Because few individuals reported having bloody diarrhoea, odds ratios for this case class could not be calculated. Interestingly, even when *tet*Q was present in the water, as the level of *E. coli* contamination increased above the first quartile, no cases of HCGI-NBD were observed.

**Table 7:** Adjusted odds ratios for diarrhoea, HCGI-BD & HCGI-NBD associated with microbial quality groups.

Microbial Quality Group	Diarrhoea		HCGI-BD		HCGI-NBD	
	aOR ^1^ (95% CI)	(*p*)	aOR (95% CI)	(*p*)	aOR (95% CI)	(*p*)
Group 1 *(tet*Q *& 1st Quartile E. coli)* ^†^	2.95 (1.35–6.45)	0.005 ^*^	N/A ^2^	-	5.31 (1.72–16.35)	0.001 ^*^
Group 2 *(tet*Q *& 2nd Quartile E. coli)*	2.06 (0.43–9.76)	0.248	N/C	-	N/C	-
Group 3 *(tet*Q *& 3rd Quartile E. coli)*	0.48 (0.03–7.27)	0.588	N/C	-	N/C	-
Group 4 *(tet*Q *& 4th Quartile E. coli)*	N/C ^3^	-	N/C	-	N/C	-

**^1^** Odds ratio adjusted for age using Mantel-Haenszel method and null and alternative hypotheses are as follows, H_0_: OR = 1 *versus* H_1_: OR ≠ 1.; ^2^ Not Available. There were only 3 cases of HCGI with bloody diarrhoea and in certain cases, ORs were not able to be calculated due to zero cell counts; ^3^ No cases exposed in this quartile; ^†^ *E. coli* concentration divided into quartiles to better demonstrate the varying risk with different contamination levels; ^*^ Statistically significant confidence intervals above an OR of 1 or “no effect”.

### 3.5. Discussion

The results from this study suggest that the greatest bacterial contamination of drinking water in the city of Maroua occurs within the household and that improvements of the originating source water quality deteriorate once the drinking water moves through the distribution system and is stored in the household. Despite the fact that a majority of the study population received their water from improved sources, 87% of drinking water samples taken from HSC surpassed the WHO’s “no action required” threshold for *E. coli* in drinking water. The *tetQ* positive samples also confirm that tetracycline-resistant bacteria are present within the study area, a steadily increasing trend in the developed and developing world [22,23]. *tet*Q had an interesting relationship between *E. coli* levels in HSC and this relationship provided a more demonstrable association with reported diarrhoeal disease than *E. coli* levels alone. This finding implies that adding additional screening factors, such as detection of antibacterial resistance genes, may provide improved assessment of the diarrhoeal risk associated with a drinking water source especially in tropical and semi-tropical regions of the world, like Maroua, where the traditional faecal indicators may not be as effective signalers of potential faecal contamination due to increased persistence of environmental presence [35,36,37].

This *tet*Q-*E. coli* relationship suggests that the growing problem of antibiotic resistant bacteria adds a new dimension to the kind of drinking water contamination that can occur. Antibiotic use is often uncontrolled in developing countries [38,39,40,41] leading to great concern as the number of organisms resistant to multiple types of antibiotics grows and is present in the greater environment. A study in Nepal demonstrated that even communal exposure to antibiotics increased the number of antibiotic resistant pathogens within an individual’s faeces [42]. In addition, animal studies have demonstrated that antibiotic use can lower the infectious dose of certain pathogens [43].

The presence of *tet*Q and of HF183 in drinking water is an interesting finding for two reasons. First, both genetic markers confirm that there is human faecal contamination (and/or possibly livestock in the case of *tet*Q) occurring within areas where drinking water is accessed and utilized in our study area. In addition, the near equal HF183 positive detection (Table 4) percentage among home and source samples and the higher *tet*Q positive detection in sources samples suggest that this specific type of contamination is coming from the greater environment and/or in addition to within the home. *tet*Q and HF183 were not found together in any of the samples, suggesting that *tet*Q genes could be potentially coming from non-human sources (*i.e.*, livestock) within the environment. The detection of *Campylobacter* spp. also supports the possibility that contamination is coming from livestock or wildlife within the home and within the areas where drinking water is collected. In the urban environment of Maroua, livestock density is relatively high (specifically goats, sheep, and poultry) and animals mingle freely within homes, on the streets and frequently congregate near public stand taps (Figure 1). Lizards and birds are also common within household compounds.

Second, *tet*Q was detected in source samples at more than twice the positive percentage found in HSC samples, suggesting that tetracycline-resistant bacteria are present within the city distribution system (from either human or livestock sources) despite chlorinated water treatment. It is possible that the bacteria with this resistance are less susceptible to disinfection or that they are being introduced at the point of collection and there is not sufficient residual chlorination remaining to kill them.

The inverse relationship between *tet*Q and *E. coli* within *canaries*, in addition to the results of the second round of odds ratios, suggests that there may be an interaction between antibiotic-resistant bacteria and *E. coli* within drinking water that has a relationship to gastrointestinal illness. While the study design did not allow for identification of which bacteria were in the water when individuals became ill, the cause of the diarrheal illness, or if individuals were currently taking antibiotics (*i.e.*, tetracycline), it seems that in our study area that tetracycline usage has caused an additional environmental risk factor to drinking water quality. And while there are some inherent limitations to using qPCR, namely lack of discrimination between viable and non-viable organisms and the potential for false detection within a mixed microbial community, the results of this study strengthen the suggestion that there may be a link between genetic determinants of antibiotic resistance and virulence such that fewer bacteria are necessary to create disease when resistance genes are present [44].

## 4. Conclusions

While our findings are exploratory and our sample sizes limited, our findings suggest that the use of antibiotics in humans or animals may potentiate an increased risk of gastrointestinal infections, especially for young children and infants, through increasing the selection for antibacterial resistant bacteria in the greater environment and that the likelihood of drinking water contamination in the home needs to be re-evaluated and further examined, taking into consideration how antibiotic use and zoonotic sources influence the microbial ecosystem of HSC and how interactions between microfauna affect human health.

Further work is warranted to better match the pathogenic organisms in the *canaries* with individuals within the household and to better assess the relationship between antimicrobial-resistant bacteria and other pathogens in micro-ecosystems within *canaries* and what this relationship’s impact is on drinking water quality.

## Supplementary Files

Supplementary File 1
