# Supplementary material for: Muddying the Waters: A New Area of Concern for Drinking Water Contamination in Cameroon
Source: Int J Environ Res Public Health. 2014 Nov 28;11(12):12454–72. doi: 10.3390/ijerph111212454 (PMC4276624; doi:10.3390/ijerph111212454)
Supplement: Supplementary File 1 [file ijerph-11-12454-s001.pdf]

Supplementary Information

Muddying the Waters: A New Area of Concern for Drinking Water Contamination in Cameroon

S1. Sample Processing and DNA Extraction

Bacterial DNA from membrane filters was extracted using a modified protocol in *Lee et al.* (2013) [1] with BeadBeater (BioSpec Products, In., Bartlesville, OK) and the QiAamp DNA stool kit (Qiagen, Valencia, CA). Briefly, water samples (190–790 mL) were filtered using a EMD Millipore Isopore™ Polycarbonate Membrane Filters (pore size: 0.4 μm, diameter: 47 mm) (Merck KGaA, Darmstadt, Germany). The filter membrane was transferred into a 2 mL sterile tube with 0.6 mg of 0.1 mm and 0.5 mm diameter sterilized glass beads (0.3 mg each; Biospec Products, Bartlesville, OK), and then DNA was extracted using QIAamp® DNA stool kit (Qiagen, Valencia, CA). After bead beating with a Mini-Beadbeater 96 apparatus (BioSpec Products, Bartlesville, OK) at 13,200 oscillations/min for 3:30 min, the supernatant was transferred to a new 2-ml microcentrifuge tube, followed by DNA extraction using a QIAamp DNA stool kit according to the manufacturer's instructions, and then suspended in 200 μL of elution buffer. The final eluates were used immediately or stored at −80°C until further processing.

S2. PCR Protocol for Quantification of Genetic Markers

For the quantification of human faecal contamination (HF183), antibacterial resistance genes (tetQ), *Campylobacter* spp. , and *Staphylococcus* aureus (*S*. aureus) in drinking water, all experiments were conducted in duplicate using the ABI 48-well StepOneTM real Time System (Applied Biosystems, Foster City, CA) with different diluted qPCR templates. Because the presence of PCR-inhibiting materials were observed in the some samples, PCR templates were diluted to mitigate potential PCR inhibition [2]. TaqMan-based real-time quantitative polymerase chain reaction (qPCR) was performed targeting HF183 and *Campylobacter* spp. and SYBR Green qPCR analysis was used for tetracycline-resistant gene (*tet*Q) and *S*. aureus [3]. The total volume of qPCR mixture was 20 μL containing 2 μL DNA template, 10 μL TaqMan Universal PCR Master Mix or SYBR Green PCR Master Mix (Applied Biosystems, Foster City, CA, USA), 500 nM primers, and 250 nM Probe (TaqMan assay only). A mixture of all PCR regents with nuclease-free water (Fisher Scientific, Fair Lawn, New Jersy, USA) was used as a negative control for each PCR reaction. PCR cycling conditions for the TaqMan analysis; an initial cycle at 50 °C for 2 min and 95 °C for 10min, followed by 40 cycles of denaturation at 95 °C for 15 s, annealing and extension at 60 °C for 1 min (HF183) [1] or at 58 °C for 30 s (*Campylobacter* spp.) [4]. The PCR protocol of SYBR green analysis for *tet*Q consisted of an initial cycle of 95 °C for 10 min, followed by 40 cycles of denaturation at 95 °C for 30 s and annealing and extension at 62 °C for 1 min. After amplification, melting curve analysis was performed by heating samples to 95 °C for 30 s, cooling them to 62 °C for 1min, and then heating them to 95 °C at a rate of 0.3 °C/s. [5]. For the quantification of *S*. aureus, the SYBR green protocol included an initial step of 95 °C for 15 min, followed by 40 cycles of denaturation at 95 °C for 15 s and annealing and extension at 60 °C for 30 s. After amplification, melting curve analysis was

performed by heating samples to 95 °C for 30 s, cooling them to 62 °C for 1min, and then heating them to 95 °C at a rate of 0.2 °C/s [3,6].

References

1. Lee, C.S.; Lee, J. Application of host-specific source-tracking tools for rapid identification of fecal contamination in fresh produce by humans and livestock: Detection of fecal contamination in lettuce with host-specific qPCR assays. *J. Sci. Food Agric.* **2013**, *93*, 1089–1096.
2. Haugland, R.A.; Siefring, S.C.; Wymer, L.J.; Brenner, K.P.; Dufour, A.P. Comparison of *Enterococcus* measurements in freshwater at two recreational beaches by quantitative polymerase chain reaction and membrane filter culture analysis. *Water Res.* **2005**, *39*, 559–568.
3. Warren, D.K.; Liao, R.S.; Merz, L.R.; Eveland, M.; Dunne, W.M. Detection of methicillin-resistant *Staphylococcus aureus* directly from nasal swab specimens by a real-time PCR Assay. *J. Clin. Microbiol.* **2004**, *42*, 5578–5581.
4. Lund, M.; Nordentoft, S.; Pedersen, K.; Madsen, M. Detection of *Campylobacter spp*. in chicken fecal samples by real-time PCR. *J. Clin. Microbiol.* **2004**, *42*, 5125–5132.
5. Lee, C.; Agidi, S.; Marion, J.W.; Lee, J. Arcobacter in Lake Erie beach waters: An emerging gastrointestinal pathogen linked with human-associated fecal contamination. *Appl. Environ. Microbiol.* **2012**, *78*, 5511–5519.
6. Paule, S.M.; Pasquariello, A.C.; Hacek, D.M.; Fisher, A.G.; Thomson, R.B.; Kaul, K.L.; Peterson, L.R. Direct detection of *Staphylococcus aureus* from adult and neonate nasal swab specimens using real-time polymerase chain reaction. *J. Mol. Diagn.* **2004**, *6*, 191–196.

© 2014 by the authors; licensee MDPI, Basel, Switzerland. This article is an open access article distributed under the terms and conditions of the Creative Commons Attribution license (http://creativecommons.org/licenses/by/4.0/).
